# Aptamer-Based Biosensors for the Colorimetric Detection of Blood Biomarkers: Paving the Way to Clinical Laboratory Testing

**DOI:** 10.3390/biomedicines10071606

**Published:** 2022-07-06

**Authors:** Anna Davydova, Mariya Vorobyeva

**Affiliations:** Institute of Chemical Biology and Fundamental Medicine SB RAS, Akad. Lavrentiev Ave., 8, 630090 Novosibirsk, Russia; kuzn@niboch.nsc.ru

**Keywords:** aptasensors, colorimetric detection, blood biomarkers, point-of-care testing

## Abstract

Clinical diagnostics for human diseases rely largely on enzyme immunoassays for the detection of blood biomarkers. Nevertheless, antibody-based test systems have a number of shortcomings that have stimulated a search for alternative diagnostic assays. Oligonucleotide aptamers are now considered as promising molecular recognizing elements for biosensors (aptasensors) due to their high affinity and specificity of target binding. At the moment, a huge variety of aptasensors have been engineered for the detection of various analytes, especially disease biomarkers. However, despite their great potential and excellent characteristics in model systems, only a few of these aptamer-based assays have been translated into practice as diagnostic kits. Here, we will review the current progress in the engineering of aptamer-based colorimetric assays as the most suitable format for clinical lab diagnostics. In particular, we will focus on aptasensors for the detection of blood biomarkers of cardiovascular, malignant, and neurodegenerative diseases along with common inflammation biomarkers. We will also analyze the main obstacles that have to be overcome before aptamer test systems can become tantamount to ELISA for clinical diagnosis purposes.

## 1. Introduction

Clinical diagnostics for infectious, oncological, autoimmune, and other diseases rely on test systems based on the specific molecular recognition of certain disease biomarkers in patients’ blood. A great majority of diagnostic systems employ antibodies as analyte-recognizing elements. The wide repertoire of specific antibodies, high sensitivity of the assays, and availability of commercial diagnostic kits with straightforward, standardized protocols made ELISA a method of choice for measuring blood biomarkers. However, ELISA has several shortcomings that originate from the intrinsic properties of antibodies. Using antibodies requires strict storage and delivery conditions for diagnostic kits. Batch-to-batch variations between different lots of the same antibody or differences in the affinity and specificity of antibodies for the same antigen made by different vendors can affect the accuracy and reproducibility of the detection. The latter problem becomes especially acute in long-term studies.

At the same time, nucleic acid aptamers—short DNA or RNA fragments that bind specified molecular targets due to a unique spatial structure—represent a prospective alternative for protein antibodies (Table 1). Owing to their chemical nature, aptamers are stable to thermal denaturation, possess a much longer shelf-life, and have no strict requirements for delivery and storage. The standard chemical synthesis of oligonucleotide aptamers guarantees minimal batch-to-batch variations. Furthermore, the in vitro selection of aptamers takes place on a lab bench and does not require the immunization of animals; therefore, aptamers can be readily selected even for non-immunogenic or toxic targets.

Currently, a large number of aptamer-based analytical systems (aptasensors) have been proposed for food safety, environmental monitoring, and the diagnosis of various diseases [1,2,3,4,5,6,7,8,9]. The relative ease of the chemical modification of aptamers and their compatibility with different biosensor platforms has provided a wide spectrum of detection systems, from portable devices to very complex sensors. The overwhelming majority of them are aptasensors with optical (colorimetric, fluorescent, or luminescent) [10,11] and electrochemical types of detection [12,13]. It should be noted that aptasensors utilizing fluorescent and electrochemical detection usually possess a high sensitivity and selectivity, but often need additional sample pre-processing, specialized equipment, and highly qualified personnel.

Nevertheless, very few of these aptamer-based test systems have found practical applications in real clinical laboratories. In our opinion, this may be because the wide potential diversity of aptamer-compatible biosensor platforms led to the dissipation of research efforts. In contrast, the characteristics of antibodies impose a greater number of restrictions. This factor limits a choice of variants for diagnostic test systems and allows for more in-depth concentration on each of them, which ultimately leads to practical use. Moreover, aptamer-based tests often represent quite sophisticated systems of an unconventional format, with equipment and protocols that are unusual for a clinical laboratory. Therefore, they are poorly perceived by the medical community, who are the end users of any diagnostic assay.

In the context of clinical diagnostics, colorimetric aptasensors have attracted particular attention. They require only a standard spectrophotometer or colorimeter, which is routine for any clinical lab, and imply typical ELISA protocols. At the moment, there are several aptamer-based commercially available diagnostic kits for the colorimetric detection of individual biomarkers in biological samples [14,15]. In our opinion, colorimetric aptasensors seem to be the most prospective candidates for routine laboratory diagnostics.

In this review, we will focus on a critical analysis of the currently developed aptamer-based colorimetric test systems, including their characteristics, limitations, and future prospects. Since blood biomarkers are of the utmost importance in clinical diagnostics and the monitoring of different diseases, we narrowed down the topic of this review to colorimetric aptasensors for the detection of blood biomarkers.

## 2. Aptamer-Based Biosensors: General Principles of Detection

**Figure 1:**
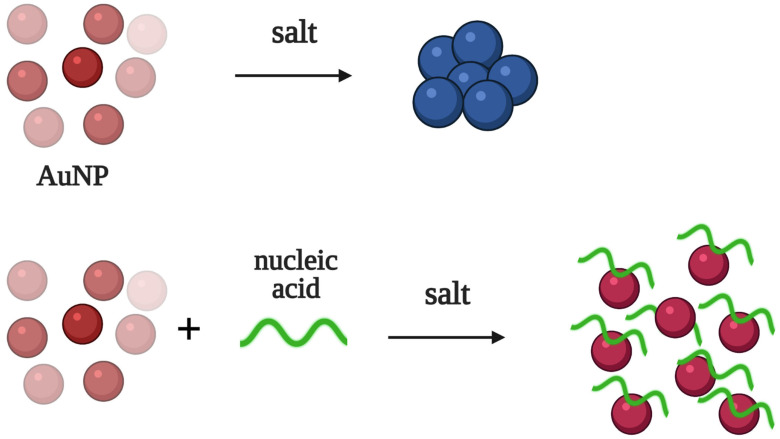
Colorimetric detection using the dispersion/aggregation of gold nanoparticles (AuNPs) in salted solution. Unmodified AuNPs aggregate in the salt-containing solution, turning a red colored solution into a blue solution. The non-specific absorption of nucleic acids prevents the aggregation of AuNPs, and the solution remains red.

Colorimetric aptasensors fall into several groups based on their principle of colorimetric signal generation. Here, we will list the most common types of detection employed in colorimetric aptasensors, which will be further discussed below. The first group of aptasensors relies on the color change provided by the dispersion and aggregation of AuNPs in the presence of different salts (Figure 1). Unmodified AuNPs tend to aggregate in salt-containing solutions, causing red to blue color changes. The non-specific absorption of polyanionic aptamers prevents the aggregation of AuNPs, and the color remains red. This type of detection is very simple, and the result can be inspected by the naked eye. At the same time, the different components of biological fluids (proteins, salts, etc.) could hinder the dispersion/aggregation of AuNPs, resulting in a lower sensitivity and selectivity of detection. 

**Figure 2:**
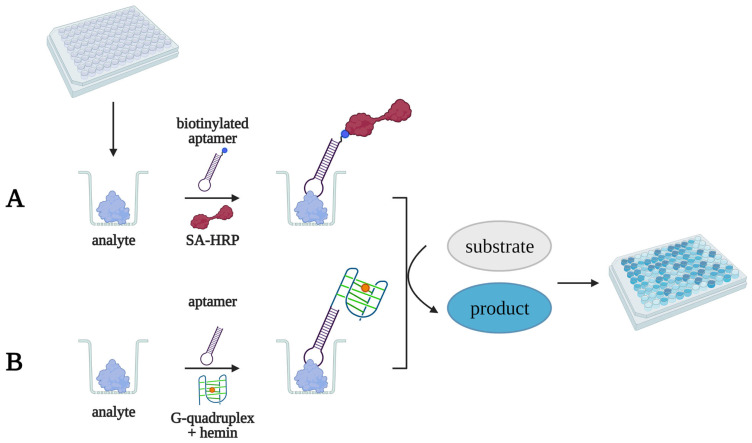
General scheme of detection for a colorimetric aptasensor with peroxidase (**A**) or peroxidase-like (**B**) generation of the analytical signal. First, a biotinylated aptamer forms a complex with the analyte in a microplate well; then, streptavidin-conjugated peroxidase binds biotin. Next, peroxidase (**A**) or a peroxidase-mimicking analog (**B**) oxidizes the chromogenic substrate, turning a colorless solution into a colored solution.

The other group of colorimetric aptasensors generates an analytical signal through an enzymatic reaction. Horseradish peroxidase and alkaline phosphatase are typically used in such aptasensors (Figure 2A). This type of test system provides a high sensitivity and selectivity and is also fully compatible with ELISA protocols and equipment. However, the properties of protein enzymes can change due to denaturation during storage or batch-to-batch variation, and this affects the reproducibility of the results.

Recently, non-covalent complexes of hemin with quadruplex-forming DNA and different nanomaterials were proposed as peroxidase-mimicking non-protein analogs (Figure 2B). This approach allows for the creation of more cost-effective and stable aptasensors.

## 3. Aptasensors for Biomarker Detection

In the present review, we concentrated on the detailed analysis of colorimetric aptasensors for the detection of biomarkers of various diseases, including oncological, cardiovascular, neurodegenerative, autoimmune, and inflammatory pathologies. When analyzing this data, we paid particular attention to the sensitivity of the test systems (limit of detection) and their selectivity (the ability to discriminate molecules similar to the target). Table 2 summarizes the characteristics of the published aptamer-based colorimetric test systems.

### 3.1. Cancer

#### 3.1.1. VEGF

Vascular endothelial growth factor (VEGF) is a signaling protein secreted by both normal endothelial and cancer cells that plays an important role in angiogenesis regulation [63]. It is now considered as an important biomarker for cancer [64], neurodegenerative diseases (Alzheimer’s disease, Parkinson’s disease, etc.) [65,66], rheumatoid arthritis [67,68], and psoriasis [69].

The selection of VEGF-binding DNA aptamers has been shown to result in quadruplex-forming aptamers with a high affinity for their molecular target [70,71]. J. Dong used a VEGF-specific DNA aptamer for colorimetric microplate sandwich-type detection [16]. In the first step, a recombinant VEGF protein was immobilized in microplate wells, with the subsequent addition of the biotinylated aptamer. Horseradish peroxidase conjugated with streptavidin was used for aptamer–VEGF complex visualization. Free VEGF in the analyzed samples bound to the aptamers in the microplate well, displacing the pre-immobilized VEGF. Surface-unbound aptamers were then washed out, and the colorimetric signal decreased with the rise in VEGF concentration. The limit of detection for the developed assay was 0.3 pM in buffer solution. This aptasensor was also used for VEGF detection in human serum samples. The obtained results agreed with the reference chemiluminescent ELISA results. Notably, this aptasensor allowed for VEGF detection in serum samples without any preliminary manipulations (filtration, precipitation, etc.), providing fast and simple detection.

**Figure 3:**
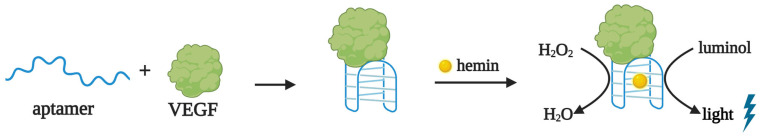
Chemiluminescent VEGF detection based on the peroxidase-mimicking activity of the hemin and G-quadruplex aptamer complex [17]. Target binding induces quadruplex structure formation in the VEGF aptamer. The resulting aptamer–target complex binds hemin and catalyzes the oxidation of luminol in the presence of hydrogen peroxide.

In general, G-quadruplex structures can bind the hemin molecule, and this complex can oxidize a chromogenic substrate in the presence of hydrogen peroxide, thus mimicking horseradish peroxidase activity. This feature of quadruplex-forming aptamers was shown to be useful for the chemiluminescent detection of VEGF [17]. After target binding, the aptamer forms an active quadruplex structure and then binds hemin; the resulting complex catalyzes the oxidation of the substrate (luminol) in the presence of hydrogen peroxide (Figure 3). In this study, the intensity of the luminescent signal linearly increased with the rise in VEGF concentration in solution. The developed aptasensor had a high sensitivity (the detection limit was 18 nM or 684 ng/mL); however, in the absence of a target, a rather high nonspecific signal appeared due to the spontaneous quadruplex formation. After dividing the aptamer into two separate oligonucleotides, the active quadruplex structure formed only in the presence of VEGF, which significantly reduced the nonspecific signal. As a result, the detection limit was lowered to 2.6 nM.

Wu et al. proposed a VEGF-specific aptasensor based on the color change in a colloid solution of AuNPs [18]. The authors designed an aptazyme consisting of the VEGF-specific aptamer and a DNAzyme connected by a short nucleotide sequence. Without a protein target, the DNAzyme and aptamer form a hairpin that prevents DNA substrate cleavage. Uncleaved DNA hybridizes with short complementary oligonucleotides on the surface of the AuNPs, thus inducing particle aggregation and a color change from red to blue. In the presence of VEGF, both the aptamer and DNAzyme are restored their active conformation. The selective cleavage of the substrate by the DNAzyme prevents the aggregation of AuNPs, and the solution remains red. The developed aptasensor was shown to detect 0.1 to 100 nM of VEGF in a buffer solution. The results of analysis in 1% spiked serum samples showed good agreement with VEGF detection in a model buffer solution, thus demonstrating the principal applicability of the assay for real clinical samples.

**Figure 4:**
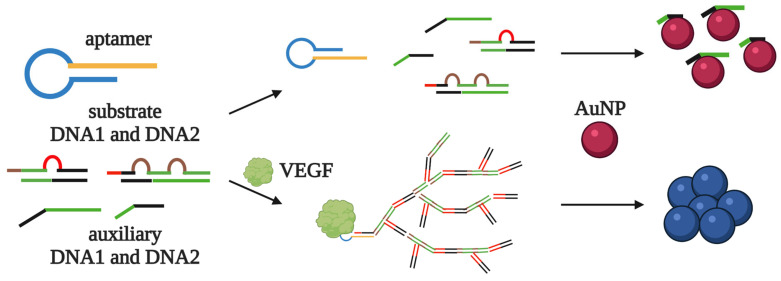
AuNP-based aptasensor for VEGF detection with signal amplification proposed by C.C. Chang [19]. The aptasensor consists of aptamer-containing hairpin DNA, two DNA substrates, and two auxiliary DNA fragments. Without the target, the single-stranded auxiliary DNA fragments are adsorbed on the AuNPs, preventing their aggregation and giving a red color to the solution. The addition of VEGF switches the aptamer to an active structure, which leads to the reorganization of the hairpin DNA. “Opened” hairpin DNA, in turn, forms a duplex with the DNA substrate and initiates a nonlinear chain reaction involving the auxiliary DNA fragments. The resulting dendrimer-like structure is poorly adsorbed on the AuNPs, and their aggregation causes a red to blue color change.

C. Chang [19] et al. proposed an even more sensitive AuNP-based VEGF detection assay with signal amplification, which takes less than an hour for the analysis and does not recruit any enzymatic reactions. The system includes aptamer-containing hairpin DNA, two DNA substrates, and two auxiliary DNA fragments. Without the target, all the DNA molecules form “closed” intramolecular structures, but not intermolecular complexes. Along with this, the auxiliary DNA fragments are adsorbed by the AuNPs, and the dispersed nanoparticles give a red color to the solution. In contrast, the addition of VEGF switches the aptamer to an active structure, which leads to the reorganization of the hairpin DNA. “Opened” hairpin DNA, in turn, forms a duplex with the DNA substrate and initiates a nonlinear chain reaction, producing the dendrimer-like structure containing auxiliary DNA fragments (Figure 4). The poor adsorption of DNA dendrimers on AuNPs leads to an aggregation of nanoparticles and a red–blue color change. The detection limit for this assay was 0.13 nM (10 ng/mL). The stabilization of AuNPs by additional oligonucleotides prevented their aggregation in the absence of VEGF and further improved the limit of detection to 185 pM (5 ng/mL). The developed assay was successfully tested on VEGF-spiked samples of diluted (2.5%) blood serum.

#### 3.1.2. CD63 as an Exosome Surface Protein

The glycoprotein CD63, a member of the tetraspanin family, is exposed on exosome membranes in different amounts depending on the cell type. Exosomes, in turn, are now considered as biomarkers of oncological diseases [72]. A DNA aptamer selected by Base Pair Biotechnologies, Inc. was used as a recognition element in different biosensors for the detection of CD63-positive exosomes. For example, Y. Jiang et al. [20] developed a colorimetric aptasensor based on the dispersion/aggregation of AuNPs, that discriminated between exosomes with different CD63 content. The authors also proposed a panel of aptamers for the simultaneous detection of several protein markers for the more accurate identification of exosomes.

**Figure 5:**
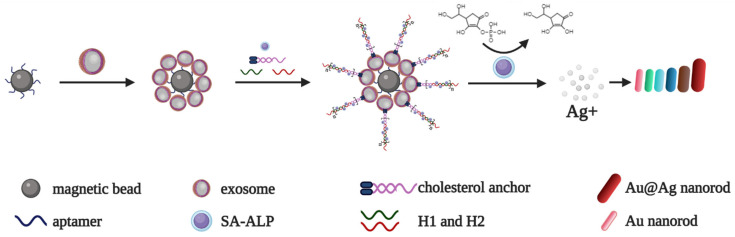
Multicolor aptamer-based system for CD63-positive exosome detection [21]. Exosomes are captured by CD63-specific aptamers immobilized on magnetic beads. Then, a cholesterol-modified DNA anchor embeds into the lipid bilayer of exosomes, with the ssDNA “sticky” end exposed to trigger a chain hybridization reaction with the biotinylated oligonucleotides H1 and H2. Next, H1 and H2 bind with streptavidin-conjugated alkaline phosphatase. The dephosphorylation of ascorbic acid phosphate in silver salt solution leads to the deposition of a silver shell on the surface of the AuNRs and a resulting multicolor change.

Zhang et al. employed the same aptamer for the multicolor detection of exosomes [21]. First, they loaded the surface of streptavidin magnetic beads with a biotinylated aptamer for the specific capture of CD63-positive exosomes. Second, a cholesterol-modified DNA probe was embedded into the lipid bilayer of exosomes, exposing the single-stranded “sticky” end to trigger a chain hybridization reaction with the biotinylated DNA oligonucleotides H1 and H2. Then, H1 and H2 formed complexes with streptavidin-conjugated alkaline phosphatase. The enzyme dephosphorylated ascorbic acid phosphate in a silver salt solution, which, in turn, led to the deposition of a silver shell on the surface of the Au nanorods (AuNRs) and a resulting multicolor change (Figure 5). The aptasensor allowed for the detection of exosomes from MCF-7 cell cultures in the range of 1400 to 280,000 particles/mL, with the limit of detection determined as 160 particles/mL. This method was also well suited for detecting exosomes from colorectal and breast cancer cell cultures, which proved its universality.

Another enzyme-recruiting colorimetric aptasensor for exosomes was proposed in [22]. First, exosomes were covalently immobilized on the surface of aldehyde latex microbeads. Then, a biotinylated CD63-specific aptamer was added to the suspension. For an analytical signal, the authors used an HRP–streptavidin conjugate and colorless dopamine solution; thus, colored polydopamine was deposited on the exosome surface. The limit of detection of the assay was 7700 particles/mL. Similar aptasensors were developed for HER2 and αvβ6 integrin detection.

Peroxidase-mimicking nanomaterials represent a promising alternative to natural protein enzymes in aptamer-based assays. For example, Y. Xia et al. [23] used single-walled carbon nanotubes (SWCNTs) with peroxidase-like activity. In the absence of a target, the CD63-specific aptamers are adsorbed on the SWCNT surface, enhancing their catalytic activity and colored product formation. In the presence of exosomes, the aptamers dissociate from SWCNTs, thus decreasing their catalytic activity. The detection limit of the assay was 5.2 × 10^5^ particles/mL. This method showed good agreement with commercial immunoassays in the analysis of serum samples from healthy donors and patients with breast cancer. A similar detection system developed in [24] recruits carbon nitride nanosheets as peroxidase mimics. The aptasensor distinguished exosomes from cancer and normal cell cultures and allowed for quantitative exosome detection in blood serum samples from breast cancer patients with a limit of detection of 13.5 × 10^5^ particles/mL.

**Figure 6:**
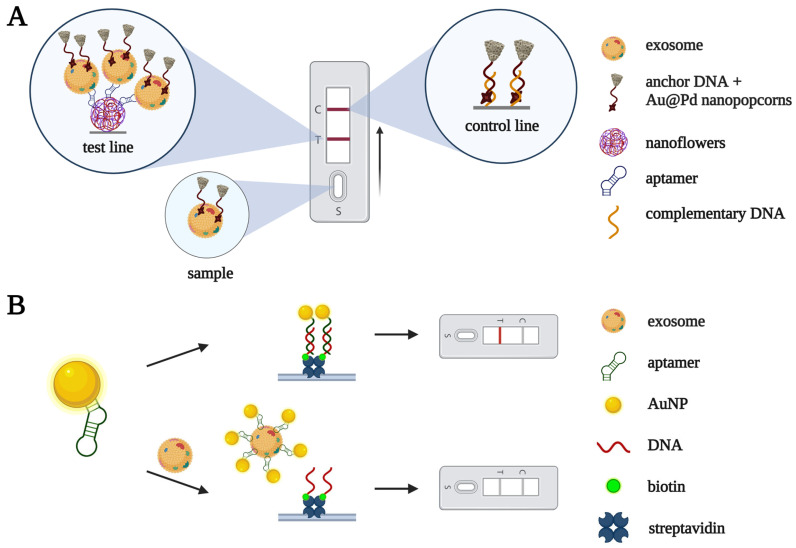
Lateral flow assays for CD63-positive exosomes. (**A**) Au@Pd nanoparticle-based aptasensor proposed in [25]. An anchor DNA fragment conjugated with Au@Pd nanopopcorn forms a complex with exosomes. Nanoflower-modified CD63 aptamers provide exosome concentration at the test line. Subsequent laser irradiation generates a thermal signal and produces a characteristic black band at the test line. (**B**) AuNP-based aptasensor developed in [26]. Without exosomes, the aptamer conjugated with AuNP binds to a complementary DNA fragment at the test line, producing a colored band due to the accumulation of AuNPs. In the presence of exosomes, the AuNP-modified aptamer binds CD63 on the exosome surface and the test line remains colorless.

Lateral flow assays for exosome detection have been constructed based on CD63-specific aptamers and nanomaterials. In [25], an anchor DNA conjugated with Au@Pd nanopopcorn captured exosomes from a solution (Figure 6A). Then, the exosome complexes were visualized using aptamer-containing nanoflowers immobilized at the test line. Specific aptamer binding with CD63 on the exosomes’ membranes caused them to become concentrated at the test line, and subsequent laser irradiation generated a thermal signal and produced a characteristic black band. The limit of detection for the assay was 1.4 × 10^4^ particles/mL, comparable with that of fluorescent and electrochemical assays. The sensitivity of detection significantly decreased in spiked serum, but the dilution of serum samples (by 10 times) improved the sensitivity.

Yu et al. [26] developed another aptamer-based lateral flow assay for exosome detection. Without exosomes, a CD63-specific aptamer conjugated with AuNPs binds to a complementary DNA fragment at the test line pad, producing a colored band due to the accumulation of AuNPs in this region. Otherwise, in the presence of exosomes, the aptamer binds CD63 on the exosome surface, and no coloring of the test line is observed (Figure 6B). This assay allowed the authors to discriminate between exosomes isolated from a non-small cell lung cancer cell culture and exosomes from a culture of fibrocytes. We should note, however, that zero signal in the presence of the analyte seems to be a serious disadvantage of the assay, making it prone to false positive results.

#### 3.1.3. Mucin-1

The transmembrane mucin glycoprotein MUC1, which is overexpressed in cancer cells, serves as a biomarker for most of the adenocarcinomas, as well as lung cancer, breast cancer, etc. [73,74].

C. Ferreira et al. selected a DNA aptamer that binds the MUC1 recombinant protein with high affinity [27]. The MUC1-5TR-1 aptamer was used as a capture probe for an ELISA-like colorimetric sandwich test system with a limit of detection of 1 μg/mL.

S. Liu et al. developed an aptazyme-based assay for MUC1 detection [28]. They combined a MUC1-specific aptamer and peroxidase-mimicking DNAzyme in one aptazyme molecule. In the absence of the analyte, the aptamer is bound to the complementary DNA immobilized on the magnetic beads and therefore can be discarded from the solution after magnetic separation. In the presence of MUC1, the aptamer forms an active structure, which induces a reorganization of the whole aptazyme molecule. As a result, the DNAzyme restores its catalytical activity and oxidizes a chromogenic substrate. The limit of detection for this aptasensor was about 5 nM, both in a model buffer solution and in 10% human serum.

Y. Zhou et al. used an aptazyme-based approach for MUC1 detection on the surface of exosomes [29]; the whole analysis took less than an hour. The limit of detection of the developed aptasensor was 3.94 × 10^5^ particles/mL.

#### 3.1.4. Carcinoembryonic Antigen

The glycoprotein carcinoembryonic antigen (CEA) is one of the most widely used biomarkers for gastrointestinal, breast, and cervical cancer [75,76].

C. Luo et al. [30] developed an aptasensor for CEA detection based on the dispersion/aggregation of AuNPs (red–blue color change). The limit of detection was 3 ng/mL in a model buffer solution. The aptasensor was tested for CEA detection in spiked samples of diluted (2%) blood serum.

K. Liang et al. also employed AuNPs in their CEA detection system with signal amplification [31]. Without CEA, an aptamer forms a duplex with complementary DNA in solution, thus blocking DNA adsorption on AuNPs. In the presence of CEA, complementary DNA dissociates from the aptamer and induces rolling circle amplification, resulting in the adsorption of single-stranded DNA fragments onto AuNPs. Measuring the absorbance ratio at 660 and 520 nm allows one to determine the CEA concentration. The limit of detection for the proposed system was 2 pM.

N. Shahbazi et al. [32] developed a homogenic assay for CEA detection with an aptazyme made of a CEA-specific aptamer and two-component quadruplex-forming DNAzyme. Two DNAzyme fragments were connected via a linker sequence complementary to the aptamer fragment. In the absence of CEA, the aptamer–linker complex inactivates the DNAzyme module. After CEA addition, the aptamer dissociates from the linker, and the DNAzyme acquires an active conformation and oxidates the chromogenic substrate. The limit of detection was 5.5 pM (1 ng/mL), and the aptazyme was successfully used for CEA detection in saliva samples from healthy donors.

#### 3.1.5. Other Cancer Biomarkers

Prostate-specific antigen (PSA) is a glycoprotein that is normally present in the blood at a very low level (0.5–2 ng/mL). An increased PSA level (4–10 mg/mL) could indicate prostate cancer [15]. Shayesteh et al. [33] developed an AuNP-based aptasensor for colorimetric PSA detection. Their assay detected PSA in the physiological range of concentrations, and the limit of detection was as low as 20 pg/mL. This method also allowed for PSA detection in spiked samples of diluted blood serum.

The transmembrane protein HER2 (human epidermal growth factor receptor type 2) is a member of the tyrosine protein kinase family. Increased HER2 expression is characteristic of lung, breast, and ovarian cancers [77]. A DNA aptamer was used for the development of colorimetric systems for HER2 detection both in solution and in LFA format [34]. Homogeneous HER2 detection based on the dispersion/aggregation of AuNPs in salted solution had a limit of detection of 10 nM in diluted (10%) serum samples. In LFA format, without a protein, the biotinylated aptamer forms a nanocomplex with AuNPs that is captured by streptavidin at the test line, resulting in the visualization of red dots. In addition, negatively charged AuNPs are trapped by a positively charged polymer at the control line, producing red coloring. In the presence of HER2, the aptamer dissociates from the AuNPs and forms a specific complex with the protein; hence, the red dots at the test line disappear, while the control line color remains the same. The limit of detection for this assay was 20 nM.

Y. Zhou et al. selected DNA aptamers that specifically bind Dickkopf-1 protein (DKK1), a WNT pathway antagonist. Increased levels of DKK-1 are typical for many types of oncological diseases [35]. The TD10 aptamer with the highest affinity was used in an ELISA-like microplate colorimetric sandwich system. The aptamer was covalently immobilized on a microplate for DKK1 capture. Anti-DKK1 antibodies served as a reporter probe to recruit streptavidin-conjugated horseradish peroxidase. The developed assay was used for DKK1 detection with concentrations ranging from 62.5 to 4000 pg/mL. The aptasensor system was successfully applied for DKK1 detection using blood serum samples from patients with colorectal cancer. The obtained results were in good agreement with the results obtained using a commercial ELISA kit.

### 3.2. Neurodegenerative Diseases

#### 3.2.1. Dopamine

Dopamine is a small organic molecule with a molecular weight of 189 Da. As a member of the catecholamine family of neurotransmitters, it has various functions in the central nervous system. Changes in dopamine levels can cause neurodegenerative pathologies, such as Parkinson’s and Alzheimer’s diseases [78]. C. Mannironi et al. selected a dopamine-binding 67 nt RNA aptamer with a K_D_ of 1.6 µM [79]. This aptamer was used in a competitive colorimetric assay for dopamine detection [36], which was analogous to competitive ELISA. The developed method was used for dopamine detection in spiked samples of diluted serum (10%) after filtration through a dialysis membrane (3 kDa). The limit of detection was 1 pM, which is about 1000 times more sensitive than ELISA.

R. Walsh et al. converted a dopamine-binding RNA aptamer into DNA form [80]. The DNA homolog retained the ability to bind dopamine with high affinity and selectivity. The limit of competitive colorimetric detection based on the DNA aptamer was 3.2 pM, close to that of RNA aptamers [37]. The DNA aptamer was used as a recognizing element in a colorimetric aptasensor based on the dispersion/aggregation of AuNPs in salt solution [38]. This aptasensor provided dopamine detection for concentrations ranging from 0.54 to 5.4 μM, and the limit of detection was 0.36 μM. 

Y. Zhang et al. used a DNA aptamer for bimodal dopamine detection [39]. Free fluorescein-conjugated aptamers in solution bind to gold nanoparticles and block their aggregation, while the particles themselves act as a fluorescence quencher for the fluorescein residue. In the absence of dopamine, fluorescently-labeled aptamers bind to AuNPs, which quench their fluorescence. In the presence of dopamine, the aptamers dissociate from the AuNPs, which leads to their aggregation and an increase in fluorescence intensity. The limit of detection was 140 nM in colorimetric mode and 78.7 nM in fluorescent mode. The proposed aptasensor was well suited for quantitative dopamine detection in spiked serum samples.

Another bimodal aptasensor for dopamine detection was developed in [40]. The DNA aptamer immobilized on the nanochip captures dopamine from solution, and a subsequent alkaline treatment results in dopamine oxidation and the formation of a colored product. The limit of detection was 0.6 μM. To further improve the sensitivity of the assay, the authors used fluorescent Au nanoclusters. In this method, a product of dopamine oxidation quenches the fluorescence of the Au nanoclusters. The limit of detection for the fluorescent assay was 3.3 nM. The principal applicability of the developed aptasensor was shown for dopamine detection in model biological samples (artificial cerebrospinal fluid and fetal bovine serum).

**Figure 7:**
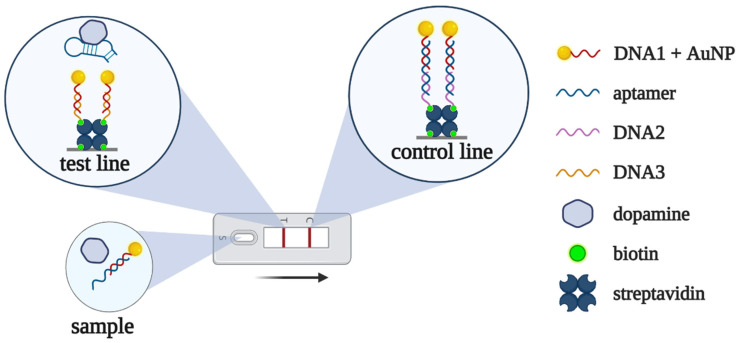
Lateral flow assay for dopamine detection proposed in [41]. Without dopamine, the aptamer forms a red-colored complex with AuNP-modified DNA1, which is trapped by DNA2 at the control line. In the presence of dopamine, the aptamer dissociates from AuNP-modified DNA1, and duplex formation between DNA1 and DNA3 provides red coloring at the test line.

N. Nakatsuka et al. [81] performed an alternative selection of dopamine-binding DNA aptamers. Their aptamer was used in a lateral flow assay to measure dopamine in urine [41]. In the absence of dopamine, the aptamer hybridizes with a complementary DNA fragment immobilized on AuNPs. The resulting complex binds to DNA at the control zone, forming a red line (zone “C” in Figure 7). In the presence of dopamine, AuNP-modified DNA dissociates from the aptamer and forms a complementary complex with another DNA, giving a red line in the test zone (zone “T” in Figure 7). The developed aptasensor was applied for dopamine detection in urine samples within clinically relevant concentration ranges (2.6–3.2 μM or 500–600 ng/mL). It is worth noting that the whole assay only took about 15 min. 

#### 3.2.2. Other Biomarkers for Neurodegenerative Diseases

Alpha-synuclein (α-syn) belongs to a group of proteins found in nerve tissue. The α-syn protein can form soluble oligomers, the increased content of which has been found in the cerebrospinal fluid and blood plasma of patients with Parkinson’s disease [82,83]. K. Tsukakoshi et al. [84] generated a DNA aptamer that selectively binds α-syn oligomers. K. Sun et al. [42] employed this aptamer to create a AuNP-based colorimetric aptasensor; however, its application for α-syn oligomer detection in real samples was restricted due to the non-selective aggregation of the AuNPs in serum.

### 3.3. Stress-Related Disease

Cortisol, a glucocorticoid hormone, participates in various physiological processes. It is considered as a biomarker of stress [85]; elevated cortisol levels are characteristic of stress-related conditions, including chronic fatigue syndrome, depression, bipolar disorder, and post-traumatic stress disorder [86,87].

J. Martin et al. selected a cortisol-binding DNA aptamer and used it as a recognizing element for a AuNP-based colorimetric aptasensor [43]. The developed aptasensor allowed for the detection of physiological concentrations of cortisol (from 150 to 600 nM). A similar aptasensor was developed by X. Bao et al. [44]. However, it was much less sensitive, and the limit of detection was only 690 μM (0.25 mg/mL).

**Figure 8:**
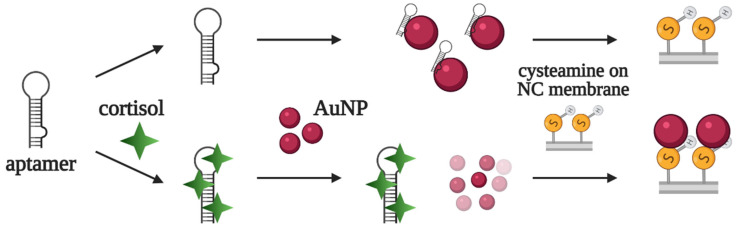
Aptamer-based lateral flow assay for cortisol detection developed in [45]. In the presence of cortisol, the aptamer binds to its target, while unbound AuNPs interact with membrane-bound cysteamine, resulting in red line formation. Without cortisol, the aptamer is adsorbed on AuNPs and prevents their interaction with cysteamine; thus, the membrane remains colorless.

The same DNA aptamer served as a component of a lateral flow assay for cortisol detection [45]. In the absence of cortisol, the aptamer adsorbs on AuNPs and blocks their interaction with cysteamine on the membrane at the test line. In the presence of cortisol, the aptamer does not bind with AuNPs; instead, they interact with cysteamine, producing a red line in the test zone (Figure 8). In contrast to most LFAs, the developed aptasensor lacks a control line on the test strip. The assay was used for cortisol detection in artificial sweat samples, with a limit of detection of 2.8 nM (1 ng/mL).

### 3.4. Cardiovascular Diseases

Cardiac troponins, troponin I and troponin T, are validated biomarkers of cardiovascular diseases, including myocardial infarction [88]. F. Torrini et al. selected DNA aptamers for troponin T and employed them in colorimetric assay [51], both in direct and sandwich formats. In the direct analysis, the biotinylated aptamer was added into microplate wells with immobilized troponin T. Then, aptamer–protein complexes were visualized using HRP–streptavidin conjugates. In sandwich format, the immobilized aptamer captures the analyte from the sample, while the second aptamer acts as a reporter. In undiluted serum samples, the limit of detection was 3.42 nM for direct analysis and 3.13 nM for sandwich format. The authors emphasized that despite the close values of the detection limits, the sandwich assay seemed to be more promising since it provided a better specific/nonspecific signal ratio.

A. Sinha et al. obtained a troponin I-specific aptamer using the on-a-chip SELEX method [89]. This aptamer acted as the analyte-capturing element of a chemiluminescent microchip aptasensor [52]. Primary troponin-specific antibodies and peroxidase-conjugated secondary antibodies were used for visualization. The limit of detection was 0.5 pM, which is comparable to commercial ELISA kits (12.5–40 pM or 300–1000 ng/L). The assay allowed for troponin I detection in blood serum samples from patients with cardiovascular diseases and from a healthy donor.

Protein HIF-1α, which controls oxygen transport, represents a potential biomarker of myocardial infarction [90]. Q. Wang et al. [53] employed the AuNP-conjugated aptamer as a reporter probe and developed a sandwich-type assay for the detection of HIF-1α on exosomes formed after myocardial infarction. Microplate-immobilized HIF-1α-specific antibodies capture the exosomes in the wells, while the peroxidase-like activity of the AuNP–aptamer conjugate provides the generation of an analytical signal. The limit of detection was 7 fM (0.2 ng/L) in a model buffer solution. The assay was applied for the detection of HIF-1α-positive exosomes in blood serum samples from model animals with myocardial infarction.

**Figure 9:**
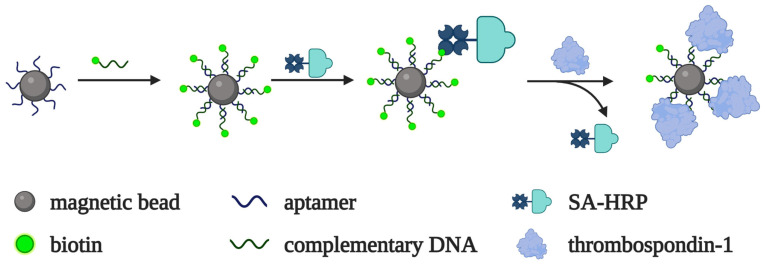
Aptamer-based system for thrombospondin-1 detection developed by K. Ji et al. [54]. The aptamer on magnetic beads forms a duplex with biotinylated DNA that binds the streptavidin–HRP conjugate. HRP oxidizes the chromogenic substrate and generates a colorimetric signal. Throbmospondin-1 displaces biotinylated DNA from the complex with the bead-bound aptamer. The peroxidase conjugate cannot bind with the beads, which leads to a decrease in the colorimetric signal intensity.

Thrombospondin-1 is a member of a family of secreted extracellular matrix proteins that play an important role in cell adhesion, migration and proliferation, angiogenesis, inflammation, atherosclerosis, and thrombosis [91,92]. A specific DNA aptamer was selected and used for the colorimetric detection of thrombospondin-1 in [54]. The aptamer, immobilized on magnetic beads, forms a complementary complex with a biotinylated oligonucleotide. In the absence of the target, the streptavidin–HRP conjugate binds to the magnetic beads due to biotin–streptavidin interactions. As the target protein displaces the biotinylated oligonucleotide from the complex with the bead-bound aptamer, the peroxidase conjugate cannot bind with the beads, which, in turn, leads to a decrease in the colorimetric signal (Figure 9). The limit of detection was 7 fM in a model buffer solution. The assay allowed for the measurement of thrombospondin-1 in blood serum samples from patients with atherosclerosis and healthy donors.

### 3.5. Other Diseases (Inflammation, Diabetes, etc.)

#### 3.5.1. C-Reactive Protein

C-reactive protein (CRP) is a general inflammatory biomarker for a wide spectrum of diseases, including cardiovascular [93] and rheumatic disorders [94]. A CRP-specific DNA aptamer was selected by B. Wu et al. for an SPR-based detection system [95]. Although the assay was very sensitive (limit of detection of 10 pM in model buffer), SPR analysis is not a common method for routine clinical diagnostics. The same aptamer served as a reporter probe for colorimetric sandwich type detection in [46]. A conjugate of the CRP-specific ligand citicoline with BSA provided the selective capture of CRP in microplate wells. A peroxidase-mimicking AuNP–aptamer complex provided CRP visualization. The limit of detection for the proposed assay was as low as 0.07 pM. The developed aptasensor allowed for the measurement of CRP in blood samples from rats with acute myocardial infarction. The results were in good agreement with those obtained using a standard ELISA kit.

M. António et al. [47] developed another AuNP-based colorimetric aptasensor for CRP detection. Without a target protein, the aptamer interacts with AuNPs and prevents their aggregation in salt solution. The addition of CRP leads to the formation of an aptamer–CRP complex and the aggregation of AuNPs, resulting in a color change. The limit of detection was 10 nM in the model buffer solution. However, the presence of serum albumin, even at a concentration 10-fold lower than that in blood (≥3 g/L), inhibited the CRP-specific dispersion/aggregation of AuNPs, which resulted in a very low sensitivity of the assay, making it inapplicable for biological samples.

#### 3.5.2. Interleukins and Their Receptors

The soluble form of the α-subunit of the IL-2 receptor, sIL-2Ra, is found at elevated levels in the sera of subjects suffering from various inflammatory processes, including autoimmune, oncological, and infectious diseases [96]. J. Jeon et al. [48] proposed a colorimetric aptasensor based on an sIL-2Rα-specific DNA aptamer. In the absence of sIL-2Rα, the aptamer–AuNP complex has an increased negative charge that attracts a positively charged substrate, orthophenylenediamine. Due to the peroxidase-like activity of AuNPs, substrate oxidation results in the development of a brown color. In the presence of sIL-2Rα, the aptamer dissociates from the AuNPs, thus decreasing the negative charge on the AuNPs, which is followed by the fading of the brown color. The developed method allowed for an express analysis (about 25 min) to be performed with a limit of detection of 1 nM, both in a model buffer solution and diluted serum samples.

Interleukin-6 (IL-6) is a cytokine involved in the immune response in various inflammatory diseases, as well as in the regulation of metabolic and regenerative processes [97]. A pair of DNA aptamers that bind different epitopes of murine IL-6 were used for a colorimetric assay based on the AuNP dispersion/aggregation effect [49]. The assay provided IL-6 detection for concentrations ranging from 1 to 125 μg/mL and took as little as 5 min for signal generation. However, this test system selectively detects only murine IL-6 and is not suited for human IL-6.

#### 3.5.3. Human Neutrophile Elastase

Human neutrophil elastase (HNE) belongs to the class of serine proteases and participates in the immune response to various pathogens. Changes in HNE expression can lead to the development of acute respiratory distress syndrome, chronic obstructive pulmonary disease, cystic fibrosis, acute lung injury, arthritis, emphysema, and atherosclerosis [98]. An HNE-specific DNA aptamer [99] was applied for colorimetric detection using the intrinsic enzymatic activity of HNE for the generation of an analytical signal [50]. The aptamer, immobilized on a solid support (magnetic particles or microplate wells), captures HNE from solution. The selective cleavage of the peptide substrate by HNE results in the generation of a colored product. The limit of detection in a model solution was 0.4 pM. However, the components of the biological samples significantly inhibited elastase activity and decreased the sensitivity of detection. While in model HNE-spiked samples, this problem was solved via the heat inactivation of inhibitors; the applicability of the assay for real clinical samples remains questionable.

#### 3.5.4. Biomarkers of Diabetes

Diabetes mellitus is a group of endocrine pathologies characterized by elevated blood glucose levels. Diabetes-related complications include cardiovascular diseases, renal failure, blindness, and foot/leg amputation [100]. The level of glycated hemoglobin HbA1c in blood provides an accurate estimation of average blood glucose for the preceding 2–3 months [101]. H. Lin et al. selected an HbA1c-specific DNA aptamer using SELEX on microchips [102]. This aptamer served as a selective capture probe for a chemiluminescent aptamer-antibody sandwich assay [55]. The aptasensor showed a limit of detection of 0.1 mM in diluted blood samples and allowed for the analysis to be performed in 25 min in automatic mode. As a further optimization of the assay, J. Li et al. [56] replaced the reporter anti-HbA1c antibody with a second DNA aptamer. The results for blood HbA1c measured by the developed aptasensor were in good agreement with those obtained using the reference HPLC method.

Different research groups [103,104,105] have performed alternative selections of hemoglobin-binding DNA aptamers. However, these aptamers have been further used as biospecific elements for electrochemical, SPR, and fluorescent aptasensors with quite complicated analytical schemes and equipment [104,106,107,108,109], which can hardly be applied in routine clinical lab practice.

Measuring the key diabetic hormone insulin also provides important information for the diagnostics and management of diabetes. A. Rafati et al. [58] applied a quadruplex-forming DNA aptamer for colorimetric insulin detection. The biotinylated aptamer was immobilized on a streptavidin magnetic bead/DNA nanotube composite. In the presence of insulin, the aptamer forms the quadruplex structure that binds hemin for the peroxidase-like oxidation of a chromogenic substrate. The limit of detection for the assay was 2.6 pM, which is comparable to an ELISA kit (42 pM).

**Figure 10:**
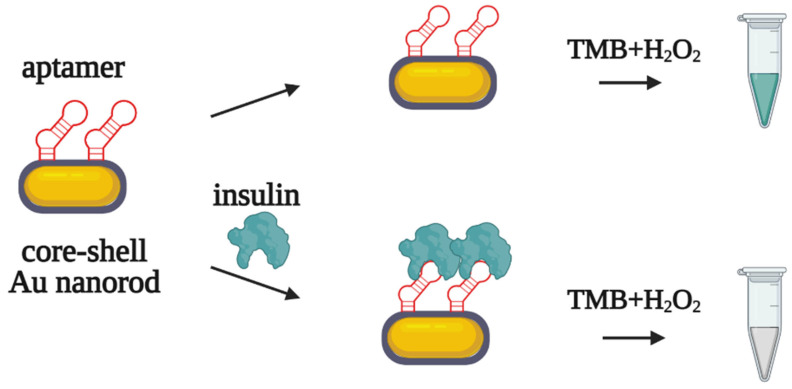
Insulin detection by aptasensor based on Au nanorods with peroxidase-like activity [59]. Without insulin, AuNRs catalyze the oxidation of the chromogenic substrate (TMB) in a peroxidase-like manner, resulting in a color change in the solution. Insulin binds with the aptamer on the AuNRs and inhibits the oxidation of TMB.

The same DNA aptamer was used in another assay for insulin detection in serum samples from patients with diabetes [59]. A thiol-modified aptamer was covalently immobilized on golden nanorods (AuNRs) possessing peroxidase-mimicking activity. In the presence of insulin, the aptamer–insulin complex inhibits the catalytic activity of AuNRs (Figure 10), while in the absence of the analyte, peroxidase-like oxidation provides a colorimetric signal. The limit of detection for the assay was 0.2 pM in serum samples. For the simultaneous detection of insulin and glucose, the analytical system was supplied with glucose oxidase. After enzymatic glucose oxidation, hydrogen peroxide accumulates in the solution and participates in the AuNR-catalyzed oxidation of the substrate. The authors suggested this binary aptasensor could be particularly useful for the differential diagnosis of type 1 and type 2 diabetes.

Adipokines, peptide hormones produced by adipose tissue, are considered as potential biomarkers of obesity and diabetes. Lee et al. [60] developed an aptamer-based microplate detection system for vaspin, visfatin, and retinol-binding protein 4 (RBP4). Adipokine-specific DNA aptamers immobilized in microplate wells were used for analyte capture, while specific antibodies were used as reporter components. The limits of detection were 3.7 nM for RBP4, 1 nM for vaspin, and 0.4 nM visfatin, both in model buffer solution and in diluted serum samples.

**Figure 11:**
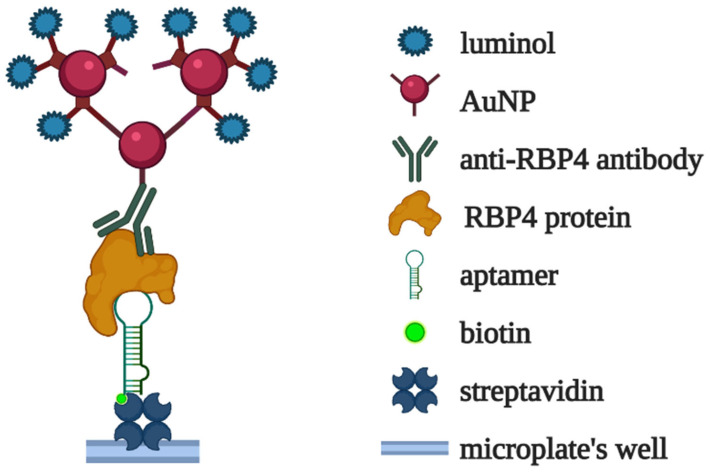
Chemiluminescent detection of RBP4 proposed in [61]. RBP4-specific aptamer captures the analyte in the microplate well. The aptamer–RBP4 complexes are visualized via conjugates of anti-RBP4 antibodies with covalently crosslinked luminol-modified AuNPs.

R. Torabi et al. [61] developed a chemiluminescent assay for RBP4 based on a specific DNA aptamer. The immobilized RBP4 aptamer selectively captured the analyte from solution. The complexes were then visualized using anti-RBP4 antibodies conjugated with covalently crosslinked luminol-modified AuNPs (Figure 11). The limit of detection for the assay was 50 fM (1 pg/mL) in a model buffer solution. The aptasensor was also applied for the measurement of RBP4 in serum samples from patients with diabetes and healthy donors, and the results agreed quite well with those obtained using a commercial ELISA kit.

**Figure 12:**
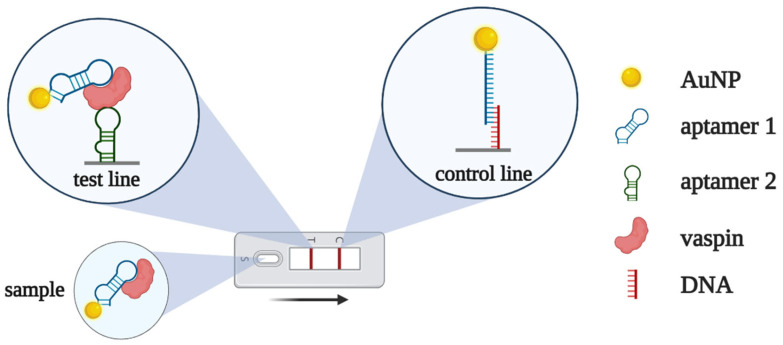
Lateral flow assay for vaspin detection based on two aptamers [62]. The complex of vaspin and AuNP-modified aptamer 1 binds with aptamer 2 at the test line, resulting in an increase in AuNP concentration and red coloring. In the absence of vaspin, AuNP-aptamer 1 binds the complementary DNA and provides red coloring at the control zone.

A pair of DNA aptamers recognizing the different epitopes of vaspin were used for detection in lateral flow assay [62]. The first aptamer—conjugated with AuNPs—served as a reporter probe, while the second aptamer—immobilized on a test line—captured vaspin from the solution (Figure 12). Without vaspin, the AuNP–aptamer conjugate binds with complementary DNA at the control line, forming a red color. In the presence of the analyte, the vaspin-bound AuNP–aptamer conjugate passes the control line and stops in the test zone, where vaspin binds to the second aptamer. The limit of detection for the proposed LFA was about 0.1 nM, both in the model buffer solution and serum samples.

## 4. Challenges and Future Directions

An analysis of the up-to-date literature shows quite a large variety of aptamer-based assays for the colorimetric detection of disease biomarkers in blood. The majority of these works are the ‘proof-of-principle’ type, which develop a general scheme of the aptasensor and pay less attention to its routine use for the analyses of clinical samples. In our opinion, for the successful translation of aptamer-based tests to clinical diagnostics, we first need a unified, generally accepted methodology for characterizing aptamer-based assays in terms of their sensitivity and selectivity/specificity. In addition, the current design of aptamer-based detection systems often does not account for interfering substances in biological fluids. As a consequence, the analytical characteristics of the assay may worsen from model analyte solutions to real clinical samples.

We propose here the following criteria for the development of aptamer-based colorimetric assays:

- The detection method must be simple and compatible with the standard equipment, consumables, and protocols in clinical diagnostic laboratories;
- The possible adverse effects of interfering substances in clinical samples (proteins, salts, small molecules, etc.) must be evaluated during the engineering of detection systems;
- If the assay protocol includes the pre-processing of the samples, this step should be properly optimized and described in the protocol in full detail.

It is worth mentioning as a separate point the design of sandwich-type colorimetric aptasensors. Test systems of this type provide especially high specificity and selectivity, but tend to rely on aptamer/antibody pairs. Surely, the displacement of even one antibody by an aptamer would improve the reproducibility, stability, and cost of the test system. However, in the further development of aptamer-based diagnostics, we must look toward aptamer/aptamer sandwich-type assays, which are completely antibody free. This task requires novel, robust techniques for the selection of pairs of aptamers for different epitopes of the same analyte.

## 5. Conclusions

The chemical nature of oligonucleotide aptamers and their stability and flexibility in assay design make them unprecedentedly useful for engineering reliable and cost-effective test systems. We are certain that the systematic, rational design of aptasensors and the creation of unified criteria for their validation will significantly broaden their area of application in clinical diagnostics and will make aptamer-based assays as routine as PCR or ELISA.

## Figures and Tables

**Table 1:** Comparison of aptamers and monoclonal antibodies.

	Aptamers	Monoclonal Antibodies
Selection method	In vitro selection	Hybridoma technology, including immunization of animals
Synthesis method	Chemical or enzymatic synthesis	Produced using cell cultures
Limitations imposed on the target molecules	No limitations	Cannot be obtained for non-immunogenic or toxic substances
Affinity	K_d_ ≈ 0.1–100 nM	K_d_ ≈ 0.1–100 nM
Specificity	High	High
Stability	Can renaturate after heat treatment Stable during long-term storage	Irreversible denaturation after heat treatment Very sensitive to delivery and storage conditions
Immunogenicity	Not shown	High
Possibility of chemical modification	Wide	Limited

**Table 2:** Colorimetric aptasensors for the detection of disease biomarkers.

Target	Type of the Aptamer	Limit of Detection	Selectivity	Ref.
VEGF_165_	DNA	0.3 pM		[16]
	DNA	18.0 nM		[17]
	DNA	2.6 nM		
	DNA	0.11 nM		[18]
	DNA	0.13 nM		[19]
CD63	DNA			[20]
	DNA	160 exosome/mL		[21]
	DNA	7700 exosome/mL		[22]
	DNA	5.2 × 10^5^ exosome/mL		[23]
	DNA	13.5 × 10^5^ exosome/mL		[24]
	DNA	1.4 × 10^4^ exosome/mL		[25]
	DNA		Fibrocyte exosomes	[26]
MUC1	DNA	83 nM		[27]
	DNA	0.09 μg/mL		[28]
	DNA	3.94 × 10^5^ exosome/mL	Exosomes from normal liver cells (L-02)	[29]
CEA	DNA	16.7 pM		[30]
	DNA	2.2 pM		[31]
	DNA	5.5 pM		[32]
PSA	DNA	0.7 pM		[33]
HER2	DNA	10 nM/20 nM (LFA)		[34]
DKK1	DNA	2.3 pM		[35]
Dopamine	RNA	1 pM		[36]
	RNA and DNA	51 nM (RNA) 0.5 nM (DNA)	Epinephrine, norepinephrine, 3-methoxytyramine, 3,4-dihydroxyphenylacetic acid, and homovanillic acid	[37]
	DNA	0.36 μM	3,4-dihydroxyphenylalanine, catechol, 3,4-dihydroxyphenylacetic acid, homovanillic acid, epinephrine, and ascorbic acid	[38]
	DNA	0.14 μM (colorimetry) 78.7 nM (fluorescence)	Hydroquinone, glucose, ascorbic acid, L-phenylalanine, L-tryptophan, uric acid, norepinephrine, 5-hydroxytryptamine, and 3,4-dihydroxyphenylalanine	[39]
	DNA	0.6 μM (colorimetry) 3.3 nM (fluorescence)		[40]
	DNA	~0.3 μM	Cortisol, epinephrine norepinephrine, and serotonin	[41]
α-Syn oligomers	DNA	10 nM		[42]
Cortisol	DNA	150 nM		[43]
	DNA	0.7 mM		[44]
	DNA	2.8 nM		[45]
CRP	DNA	0.07 pM		[46]
	DNA	10 nM		[47]
sIL-2Ra	DNA	1 nM		[48]
IL-6	DNA			[49]
HNE	DNA	0.4 pM		[50]
Troponin T	DNA	3.13 nM		[51]
Troponin I	DNA	0.5 pM		[52]
HIF-1α	DNA	2 fM		[53]
Thrombospondin-1	DNA	7 fM		[54]
HbA1c	DNA	0.1 mM		[55]
	DNA			[56]
	2′-F-RNA			[57]
Insulin	DNA	2.6 pM		[58]
	DNA	0.2 pM		[59]
RBP4	DNA	3.7 nM		[60]
	DNA	50 fM		[61]
Vaspin	DNA	1 nM		[60]
	DNA	0.1 nM		[62]
Visfatin	DNA	0.4 nM		[60]

## Data Availability

Not applicable.
